# Kinin B1 Receptor Mediates Renal Injury and Remodeling in Hypertension

**DOI:** 10.3389/fmed.2021.780834

**Published:** 2022-01-18

**Authors:** Debargha Basuli, Rohan Umesh Parekh, Acacia White, Abdullah Thayyil, Srinivas Sriramula

**Affiliations:** ^1^Department of Pharmacology and Toxicology, Brody School of Medicine at East Carolina University, Greenville, NC, United States; ^2^Department of Nephrology and Hypertension, Brody School of Medicine at East Carolina University, Greenville, NC, United States; ^3^Department of Pathology, Brody School of Medicine at East Carolina University, Greenville, NC, United States

**Keywords:** hypertension, kinin B1 receptor, renal fibrosis, inflammation, oxidative stress

## Abstract

Despite many readily available therapies, hypertensive kidney disease remains the second most prevalent cause of end-stage renal disease after diabetes, and continues to burden patient populations and escalate morbidity and mortality rates. Kinin B1 receptor (B1R) activation has been shown to have a role in the development of hypertension, one of the major etiologies for chronic kidney disease. However, the role of B1R in hypertension induced renal injury and remodeling remains unexplored. Using a DOCA-salt-induced hypertensive mouse model, we investigated whether B1R deficiency reduces hypertensive renal injury and fibrosis. To further recognize the translational role of B1R, we examined the expression of B1R and its correlation with collagen deposition in renal biopsies from control and hypertensive kidney disease patients. Our data indicates that renal B1R expression was upregulated in the kidneys of DOCA-salt hypertensive mice. Genetic ablation of B1R protected the mice from DOCA-salt-induced renal injury and fibrosis by preventing inflammation and oxidative stress in the kidney. Cultured human proximal tubular epithelial cells expressed B1R and stimulation of B1R with an agonist resulted in increased oxidative stress. In human kidney biopsy samples, we found that the B1R immunoreactivity was not only significantly increased in hypertensive patients compared to normotensive patients, but also there is a positive correlation between B1R expression and renal fibrosis levels. Taken together, our results identify a critical role of B1R in the development of inflammation and fibrosis of the kidney in hypertension.

## Introduction

Chronic kidney disease (CKD) has become a mounting problem as its prevalence continues to grow at an alarming rate, and as of 2021 it affects more than 1 in 7 adults (15%) in the US according to Centers for Disease Control and Prevention. Additionally, CKD is one of the most prevalent comorbidities in adults with diabetes (1 in 3 adults have CKD) and hypertension (1 in 5 adults). Hypertensive kidney disease is one of the leading causes of CKD. Along with high blood pressure, some of the most common risk factors associated with CKD are race, preexisting kidney damage, genetics, and aging (1). Interestingly, the risk of hypertensive kidney disease persists even after adequate blood pressure control, particularly in African Americans when compared to Caucasians (2, 3), indicating a genetic role. More recent studies have suggested an association with certain genes and metabolic pathways (4), complement activation (5), proton channel mediated reactive oxygen species (ROS) production (6), and inflammation (7) as reasons for these disparities. The pathogenesis of hypertensive kidney disease is multifactorial, and current therapeutics have relieved some patients of their condition. However, many patients still suffer from elevated blood pressure and end organ damage, despite therapeutic intervention, which further emphasizes why new treatment options are needed.

The renin-angiotensin-aldosterone system (RAAS) is a well-known regulator of blood pressure and renal fibrosis that has been widely targeted by different antihypertensive agents including renin inhibitors, aldosterone inhibitors, angiotensin converting enzyme inhibitors (ACEi), and angiotensin receptor blockers (ARBs) to control hypertension and the progression of hypertensive nephrosclerosis (HNS) (8). Although commonly used, in the clinical setting, ACEi/ARBs have been restricted in patients with a history of angioedema, bilateral renal artery stenosis, and hyperkalemia along with reproductive women not practicing contraception and when there is a >30% decline in GFR after starting. Furthermore, ~30–50% of patients taking ACEi experience a phenomenon known as aldosterone breakthrough or aldosterone escape that may not only attenuate the clinical benefit of RAAS blockade but also may result in clinical deterioration of the kidney (9, 10). Thus, while the use of ACEi and ARBs has provided a benefit in hypertensive kidney disease, new therapeutic approaches targeting different pathways of pathogenesis are critically needed.

The kallikrein-kinin system (KKS) is composed of a family of vasoactive proinflammatory peptides that mediate an extensive range of physiological actions and has recently emerged as a novel therapeutic target in controlling blood pressure (11–14). The physiological actions of kinins are mediated by two subsets of G-protein coupled receptors: bradykinin or kinin B1 receptor (B1R) and kinin B2 receptor (B2R). While the B2R is constitutively expressed in the body, the B1R is not expressed under basal conditions but is rather upregulated in the presence of inflammation and tissue damage (15). Previous studies have shown that B1R gene expression is upregulated in renal tissues of hypertensive animals (16–18). A previous study showed an association between B1R protein expression and inflammation in renal biopsies of patients with glomerulonephritis, and that B1R blockade reduced renal chemokine expression and macrophage accumulation in a murine immune model of progressive glomerulonephritis (19). In addition, B1R has also been shown to be upregulated in peripheral monocytes in humans, leading to target-organ damage within patients with essential hypertension (20).

Over the course of the last decade, multiple studies using kidney transplantation models have supported the credence behind the essential functions of the kidney in the pathogenesis of hypertension (21, 22). Our laboratory previously showed that B1R contributes to the development of neurogenic hypertension in mice, offering a novel pathway for the pathogenesis of hypertension (13). However, the role of B1R in hypertension induced renal injury remains unexplored. Therefore, we further investigated the role of B1R in the kidney with hypertension in this study. The DOCA-salt hypertension model serves as a well-recognized model of moderate to severe salt-sensitive hypertension and renal dysfunction due to its effects on imbalanced renal sodium handling as a result of an incorporated high-salt diet and uninephrectomy (23, 24). Together, these findings provide a strong model to study the role of B1R in the pathogenesis of HNS. With our previous findings and supporting published literature, we have further elucidated the role of the KKS through B1R in the pathogenesis of hypertensive kidney disease. In this study, we determined whether B1R mediates kidney injury in a low renin, salt- sensitive model of hypertension.

## Methods

### Animals

The mice were housed in a temperature (23 ± 1°C)- and humidity-controlled facility under a 12-h dark/light cycle, fed standard mouse chow (Prolab IsoPro RMH 3000, #3005737-220, Lab Diet) and water ad libitum. The experiments were performed on adult male mice (12–16 weeks old). Bradykinin B1 receptor knockout (B1RKO) mice were a generous gift from Dr. Michael Bader (Charité Hospital, Berlin, Germany) and originated from the backcrossing of an initially mixed genetic background (129/Sc and C57Bl/6) with C57Bl/6 mice (13). Wildtype (WT) C57Bl/6NJ mice (stock no. 005304) were purchased from the Jackson Laboratory. All animal studies were approved by the East Carolina University Animal Care and Use Committee (AUP #W254) and were performed in accordance with the National Institutes of Health (NIH) Guidelines for the Care and Use of Laboratory Animals and ARRIVE guidelines.

### DOCA-Salt Hypertension Model and Physiological Recordings

For each surgery, mice were anesthetized with isoflurane (2%) in an oxygen flow (1 L/min) and placed on a heating pad to maintain body temperature. Pre- and post-operative care included buprenorphine injection to relieve pain (0.05 mg/kg, sc). The mice underwent uninephrectomy. An incision was made on the skin in the retroperitoneal region, and the right kidney was removed. After 1 week of recovery, mice were implanted with telemetry probes (TA11PA-C10, Data Sciences International) for conscious blood pressure (BP) monitoring, as described previously (13). A week later, baseline BP was recorded for 3 days. Mice were then randomly divided into 4 groups (*n* = 12/group) and implanted subcutaneously either with a DOCA-silicone sheet (DOCA group, DOCA:silicone = 1:3; DOCA 1 mg/g body weight) or an empty silicone sheet (Sham group). The mice receiving DOCA were switched to 1% NaCl in drinking water, and the sham mice received autoclaved tap water. BP was continuously recorded for 3 weeks. At the end of the protocol, mice were euthanized under anesthesia. The tissues, urine and plasma were collected, and the kidney cortex was dissected under a microscope and stored at −80°C until used for experiments.

### Human Kidney Sections

All procedures with use of human tissue sections were performed according to approved protocols by the Office of Prospective Health and Biological Safety (Biological Safety Registration #17-01; 20-01). De-identified formalin fixed, paraffin embedded kidney biopsy tissue sections from patients were obtained from the biorepository of the Department of Pathology, East Carolina University/Vidant Hospital. The sections were stained with Masson's trichrome for light microscopy examination. B1R specific antibody (#bs8675R, lot 9C20V14, Bioss) was used for immunohistochemical staining of B1R. The sections were incubated with the primary rabbit anti-B1R antibody (1:200 dilution) overnight at 4°C and developed using ImmPRESS Excel Staining Kit (MP-7601, Vector Laboratories) following manufacturer's instructions. All slides were imaged using the slide scanner Philips IntelliSite Ultra Fast Scanner. Representative images were then extracted and shown. Quantification of the collagen deposition and B1R immunoreactivity was performed using NIH ImageJ software and presented as percent area of staining. Control group (*n* = 5) was defined as patients whose blood pressure was normal and not on any antihypertensive medications. Patient group (*n* = 5) was defined as patients with CKD (stage 3–4) with history of longstanding hypertension and histologic evidence of arterionephrosclerosis with medial thickening and hyalinosis of medium-sized arteries (25).

### Cell Culture and Treatment

Human kidney proximal tubular epithelial (HK-2) cells were purchased from the ATCC (cat no. CRL-2190). HK-2 cells were cultured in Keratinocyte Serum Free Medium (K-SFM, Invitrogen #17005-042) and supplemented with 0.05 mg/ml bovine pituitary extract and 5 ng/ml human recombinant epidermal growth factor as per the ATCC recommendation. The cells were grown in a humidified incubator at 37°C in an atmosphere 5% CO_2_ and 95% air. Fresh growth medium was added to cells every 3–4 days. When cells were more than 80% confluent, plated into 6-well or 48-well culture plates, cultured for 24–48 h, and used for experiments at 70–80% confluence. HK-2 cells were stimulated with B1R agonist and antagonists as described in results.

### Immunofluorescence Staining

Anesthetized mice were perfused transcardially with PBS (0.1 M, pH 7.4) for 2 min followed by 4% paraformaldehyde in PBS (0.1 M, pH 7.4) for 10 min as described previously (13). The kidneys were harvested and post fixed for 2 h in 4% paraformaldehyde in PBS (0.1 M, pH 7.4). Frozen kidney sections (5 μm) on slides were air-dried for 10 min and blocked with blocking buffer made of 5% donkey serum in PBS containing 0.2% Tween-20 for 1 h at room temperature. Then sections were incubated with KIM-1 (ab47635, lot #GR3295411-1, abcam), Fibronectin (ab2413, lot #GR3274504-2, abcam), α-SMA (ab5694, lot #GR3183259-32, abcam), TGF-β (ab92468, lot #GR3203762-5, abcam), B1R (#ABR-011, lot An-01, Alomone labs, 1:500 dilution) antibodies at 4°C overnight. Immunolabeling was done with appropriate Alexa Fluor 488 (Donkey anti-Rabbit Alexa Fluor Plus 488, A32790 Invitrogen, 1:1000 dilution) and 594 (Donkey anti-Rabbit Alexa Fluor Plus 594, A32754 Invitrogen, 1:1000 dilution) conjugated secondary antibodies for 60 min at room temperature. Sections were mounted with ProLong Diamond Anti-Fade Mount (Invitrogen) and images were captured using a fluorescence microscope (Keyence/Echo revolve). Mean fluorescent intensity was quantified using ImageJ software (NIH) and presented as graphs.

Expression of B1R in HK-2 cells was confirmed by immunostaining. Briefly, the HK-2 cells were grown on poly-L-lysine-coated glass coverslips in 12-well plates and treated with B1R specific agonist des-Arg^10^-kallidin (DAKD) for 24 h. The cells were washed with PBS, fixed with 4% paraformaldehyde for 15 min. The cells were washed with 100 mM Glycine in 1 × PBS for 5 min each 3 times and followed by incubating with 0.1% Triton X-100 in 1 × PBS for 15 min to permeabilize cells. The cells were incubated with the anti-B1R primary antibody (#bs8675R, lot 9C20V14, Bioss, 1:250 dilution) at 4°C overnight. Cells were washed with 0.1% Triton X-100 in 1 × PBS for 10 min each for 3 times and then incubated with specific secondary antibody (Donkey anti-Rabbit Alexa Fluor Plus 488, A32790 Invitrogen, 1:1000 dilution) for 1 h at room temperature in the dark. Also, a set of HK-2 cells on glass coverslips were processed without primary antibody and incubated with only secondary antibody to serve as no primary antibody controls. After 3 washes, coverslips were counterstained with DAPI, then mounted with ProLong Diamond (ThermoFisher) antifade medium. Images were captured using a fluorescence microscope (Keyence).

### Gene Expression Analysis by Real Time qRT-PCR

Gene expression was measured using real time quantitative RT-PCR, as described previously (13). Total RNA from the kidney tissue or HK-2 cells was extracted using the Direct-Zol RNA miniprep plus kit (Zymo Research) according to manufacturer's protocol. RNA concentration was measured using a spectrophotometer (NanoDrop One). The total RNA (1 μg) from each sample was reverse transcribed into cDNA using High-Capacity cDNA Reverse Transcription kit (Applied Biosystems, #4368813) according to the manufacturer's instructions. Real Time PCR amplification reactions were performed with a Taqman Fast Advanced Master Mix (Applied Biosystems, #4444557) using a QuantStudio 6 Flex real time PCR machine (Applied Biosystems). Data were normalized to β-actin expression by the 2^−(ΔΔCT)^ comparative method and expressed as a fold change compared to control.

### Protein Expression Analysis by Western Blot

Tissue samples were homogenized in 1X tissue lysis buffer containing a protease and phosphatase inhibitors cocktail (Roche cOmplete Tablets, Mini EDTA-free, # 04 693 159 001) and incubated on ice for 15 min. Lysates were cleared by centrifugation at 12,000 g and 4°C for 15 min. After determining protein concentration using BCA protein assay kit (Thermo Fisher/Pierce), 30 μg of protein lysates were mixed with Laemmli buffer, heated at 95°C for 5 min, and cooled on ice for 3 min. The samples were resolved on 4–15% Mini-PROTEAN TGX gels (Bio-Rad) under reducing conditions and blotted on to PVDF membranes using Trans turbo system (Bio-Rad). Membranes were blocked with odyssey TBS blocking buffer (Licor) and immunoblotted overnight at 4°C with a specific antibody against B1R (#ABR-011, Alomone labs). The membranes were then incubated with the corresponding secondary antibody, and bands were visualized using the Odyssey Clx imager (Licor). The density of protein bands were quantitatively analyzed by ImageJ software (version 1.52p, NIH) and expressed as a relative ratio against the loading control.

### Determination of Reactive Oxygen Species

Reactive oxygen species (ROS) levels were determined using dihydroethidium (DHE). Freshly cut mouse kidney sections were incubated with 10 μM DHE in a light protected humidified chamber at 37°C for 15 min. The presence of superoxide radicals results in oxidation of non-fluorescent hydroethidine to fluorescent 2-hydroxyethidium which intercalates with DNA in the nuclei. Images of ethidium-stained tissue were obtained with a fluorescence microscope (Keyence). Ethidium bromide was excited at 488 nm, and fluorescence was detected at 560 nm (26, 27). Mean fluorescence intensity of the digitized image was measured with ImageJ software (version 1.52p, NIH) for quantification. The data were expressed as relative fluorescence intensity (28). In addition, the production of ROS in HK-2 cells was measured spectrofluorometrically by using the probe DHE. Briefly, the HK-2 cells were cultured in 48-well plates and treated with 10 μM DHE (Invitrogen) for 30 min. Cells were exposed to DAKD or vehicle for 30 min with or without a pretreatment of R715 or HOE-140 for 30 min. Then the DHE medium was removed, and cells were washed twice with PBS. Fluorescence was detected on a microplate spectrofluorometer (Tecan infinite m200) at an excitation wavelength of 488 nm and emission wavelength of 610 nm based on previous studies that used spectrofluorometer based detection (29, 30). Treatment with antimycin A, an inhibitor of complex III of the mitochondrial electron transport chain, is used as a positive control for ROS generation. Treatment with N-acetyl Cysteine is used as an antioxidant control. Data were expressed as total fluorescence in relative fluorescence units (RFU).

### Statistical Analysis

Data are presented as mean ± SEM. Statistical analyses were performed using GraphPad Prism 7 (GraphPad Software). Experiments involving two groups were compared using unpaired, 2-tailed *t*-tests. Multiple comparisons were made using One-way ANOVA or two-way ANOVA, followed by Bonferroni's *post-hoc* analysis or Tukey's multiple comparisons test, as appropriate. The correlation analysis of B1R expression and fibrosis was performed using linear regression analysis. Differences were considered statistically significant at *P* < 0.05.

## Results

### B1R Expression Is Upregulated in the Kidneys of DOCA-Salt Hypertension Mice

**Figure 1:**
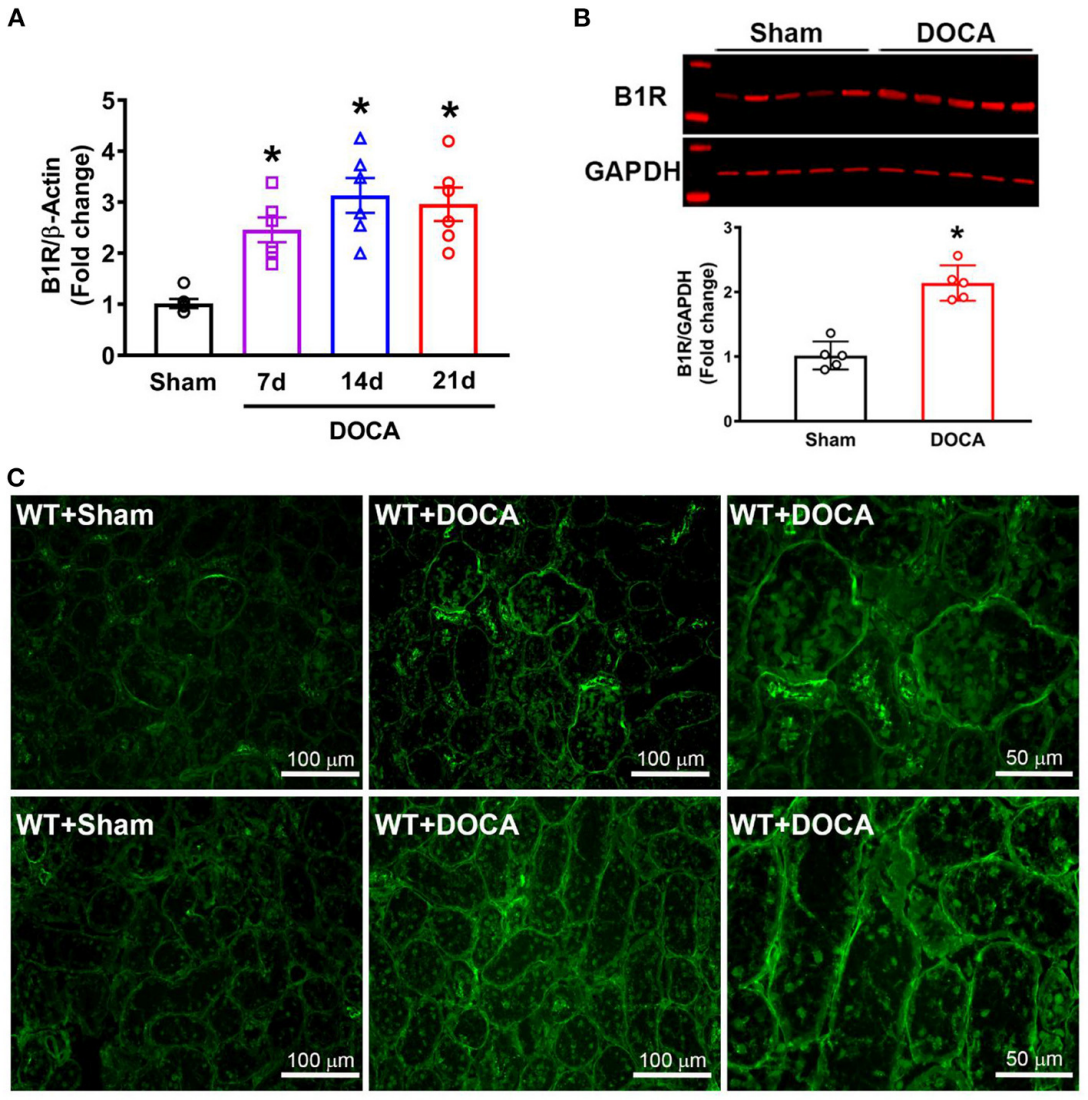
Elevated B1R expression in the kidneys of DOCA-salt hypertensive mice. **(A)** Gene expression measured by real time RT-PCR showing significantly increased mRNA in the kidneys at 7, 14, and 21 days of DOCA-salt treated hypertensive mice compared with sham mice (*n* = 6, One-way ANOVA, *P < 0.01 vs. Sham). **(B)** Representative western blot image and quantification data showing significantly increased B1R protein expression in the kidney cortex of hypertensive mice (*n* = 5, Unpaired, 2-tailed *t*-test, *P < 0.01 vs. Sham). **(C)** Immunofluorescence staining showing increased B1R expression in the glomeruli (top row) and tubules (bottom row) of DOCA-salt treated wild-type (WT) mice compared to sham treated mice (B1R, green).

First, we investigated the changes in B1R expression in the kidneys of hypertensive mice at different timepoints during the DOCA-salt treatment. B1R gene expression was significantly increased at day 7 and remained elevated throughout 3 weeks in DOCA-salt treatment compared with sham mice (Figure 1A; ^*^p < 0.01), which correlated with the increase in blood pressure (Supplementary Figure 1). Furthermore, western blot analysis showed that B1R protein expression was also significantly increased in the cortex of hypertensive mice compared to sham controls (Figure 1B; *p* < 0.01). To further visualize B1R expression within the kidney, immunostaining of kidney sections with a validated B1R specific antibody was performed, showing an upregulation of the receptor within the glomeruli and tubules of mice treated with DOCA-salt (Figure 1C).

### B1R Gene Deletion Prevents the Expression of Fibrosis Markers in the Kidney During DOCA-Salt Hypertension

**Figure 2:**
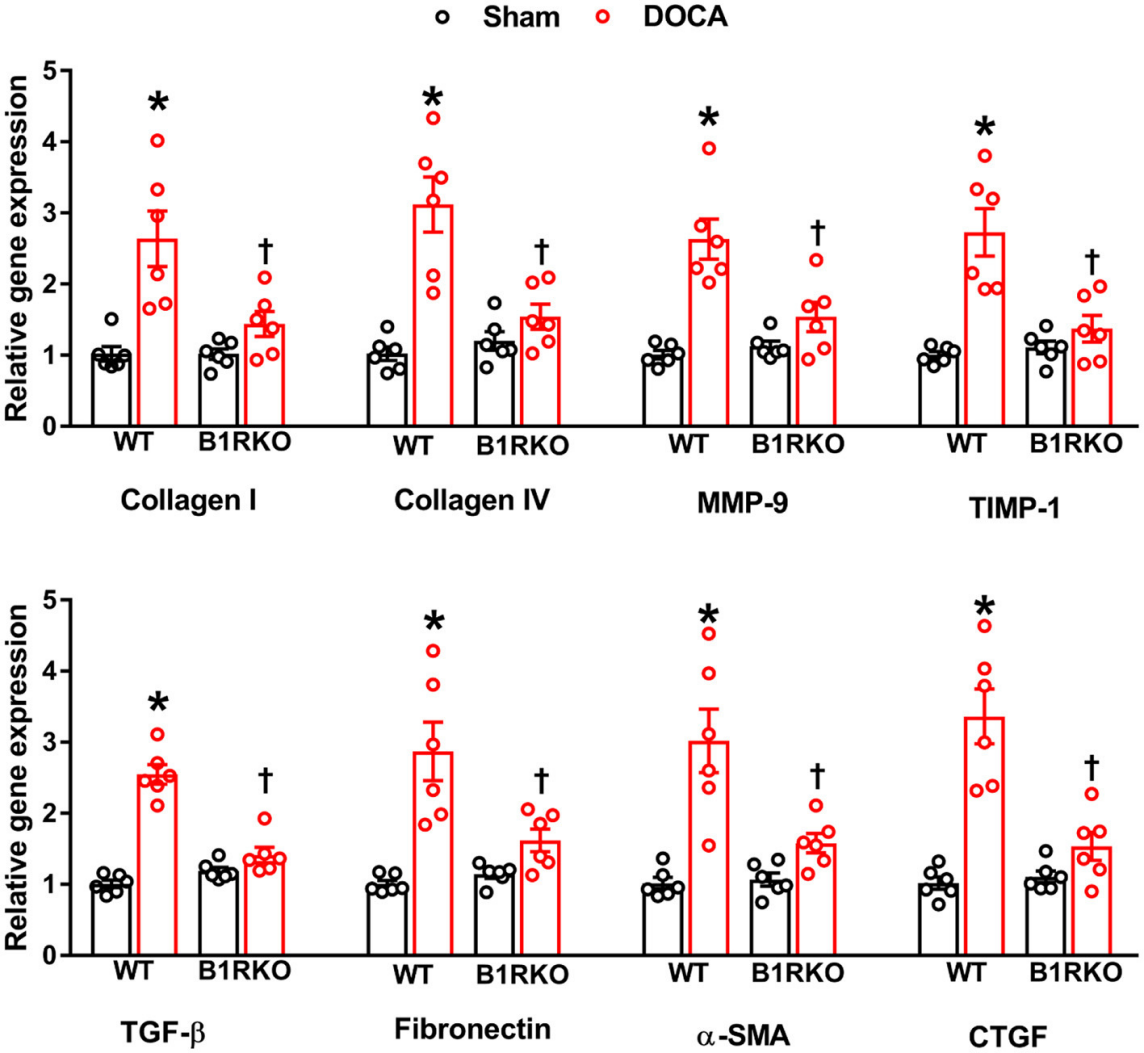
B1R gene deletion prevents gene expression of renal fibrosis markers in DOCA-salt hypertension. Gene expression measured by real time RT-PCR showing significantly increased mRNA of renal fibrosis markers in the kidney cortex of DOCA-salt treated hypertensive mice compared with sham mice. B1R knockout mice did not show this DOCA-salt-induced increase in renal fibrosis markers. Data was normalized to β-actin and presented as mean ± SEM (*n* = 6, Two-way ANOVA, *P < 0.01 vs. Sham, ^†^*P* < 0.05 vs. WT + DOCA).

Since the hallmark of progressive hypertensive kidney disease is excessive extracellular matrix (ECM) production leading to renal fibrosis, we investigated if B1R has any role in regulating renal fibrosis (31). To determine whether B1R affected renal fibrosis *in vivo*, we compared the degree of fibrosis by measuring pro-fibrotic markers in the kidneys of WT and B1RKO mice with and without DOCA-salt treatment. DOCA-salt treatment significantly increased the gene expression of fibrosis markers such as collagen (I and IV), MMP-9, TIMP-1, TGF-β, fibronectin, and connective tissues growth factor (CTGF) (Figure 2; *p* < 0.01). Additionally, α-SMA gene expression was upregulated, suggesting increased collagen turnover and extra cellular remodeling, further evidencing the potential for myofibroblast transdifferentiation – a phenomenon known to have a crucial role in the development and progression of renal tubulointerstitial fibrosis (32). In B1RKO mice, gene expression of these profibrotic markers were prevented, indicating the potential role of B1R in DOCA-salt-induced fibrosis in mice.

### B1R Gene Deletion Abrogates DOCA-Salt Induced Renal Myofibroblast Transition

**Figure 3:**
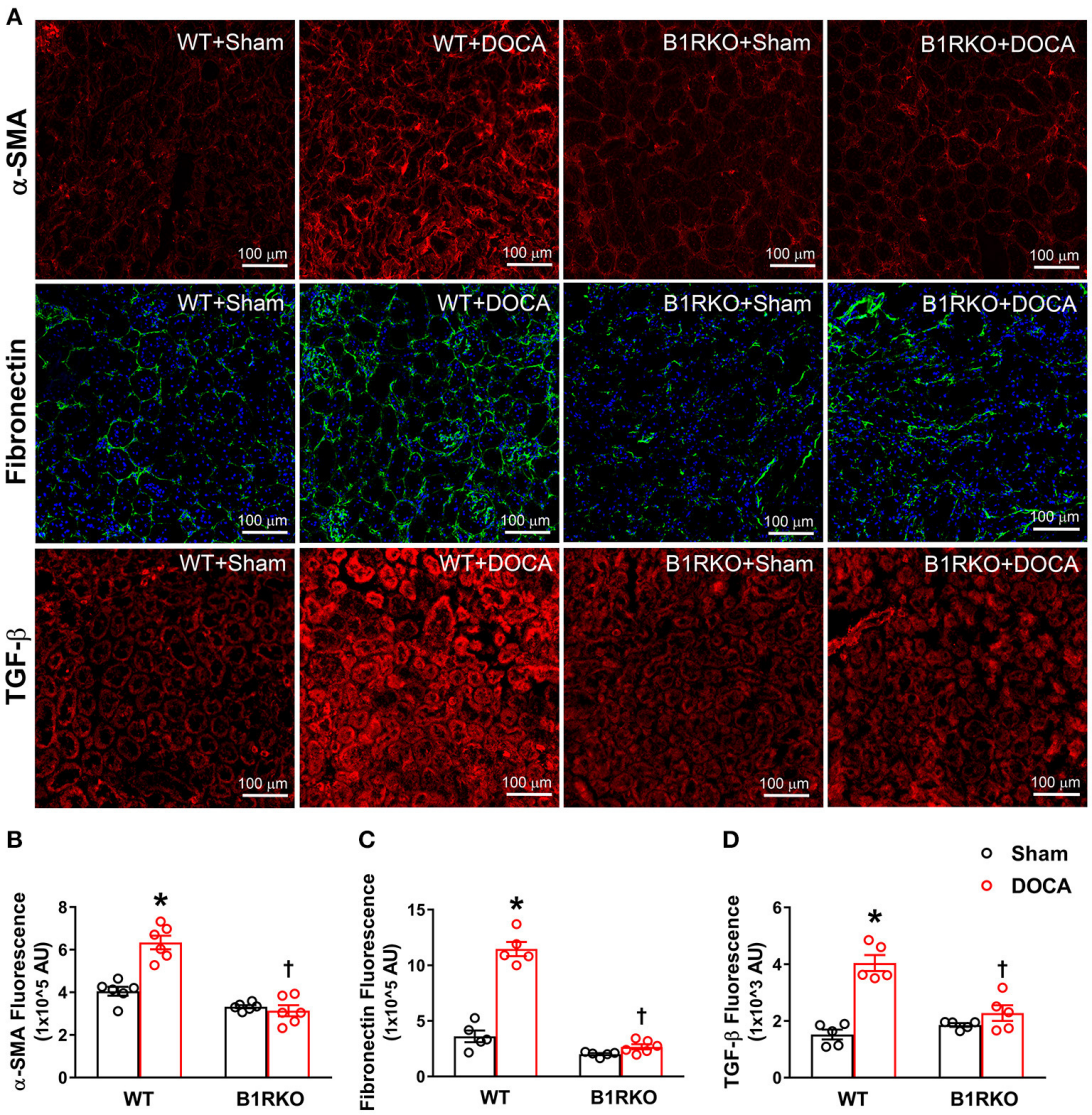
B1R gene deletion abrogates DOCA-salt induced renal myofibroblast transition. **(A)** Representative immunofluorescence images showing expression of alpha-smooth muscle actin (α-SMA), fibronectin, and transforming growth factor-beta (TGF-β) in the kidney sections. Quantification data presented as mean fluorescence staining showing increased expression of α-SMA **(B)**, fibronectin **(C)**, and TGF-β **(D)** of DOCA-salt treated hypertensive mice compared with sham mice. These increases in fibrosis markers expression were blunted in B1R deficient mice with 3 weeks of DOCA-salt treatment (*n* = 6, Two-way ANOVA, *P < 0.01 vs. Sham, ^†^*P* < 0.05 vs. WT + DOCA).

In response to stress or injury, fibroblasts transition to myofibroblasts, the activated phenotype of fibroblasts, and are responsible for ECM deposition in fibrosis. DOCA-salt treatment significantly increased α-SMA, fibronectin, and TGF-β protein expression in WT mice but was reduced in B1RKO mice (Figure 3; *p* < 0.01). This indicates that B1R may play a role in preventing DOCA-salt-induced fibrogenic activation and transition of fibroblasts into myofibroblasts.

### B1R Gene Deletion Prevents Kidney Injury During DOCA-Salt Hypertension

**Figure 4:**
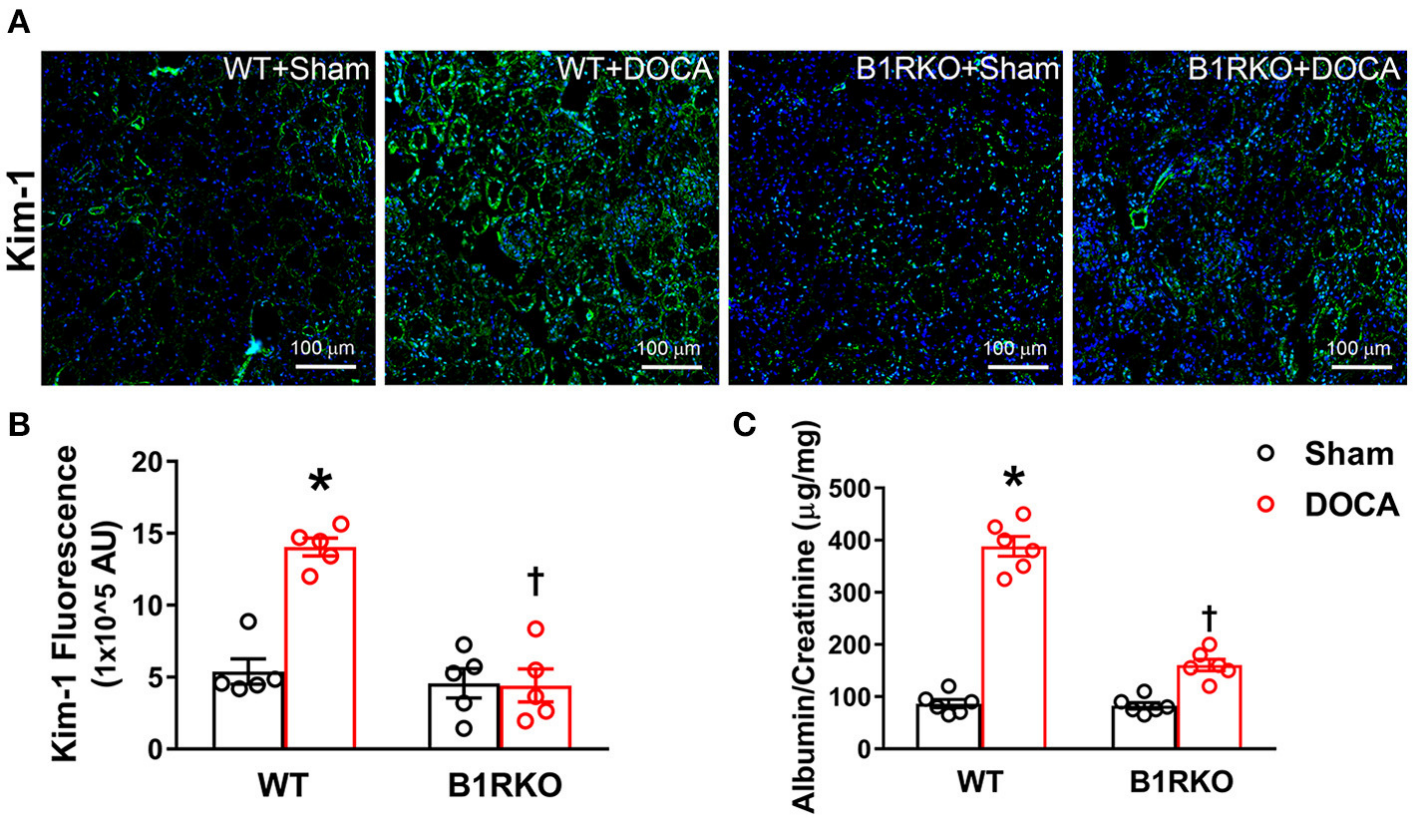
B1R gene deletion prevents kidney injury in DOCA-salt hypertension. **(A)** Representative immunofluorescence images showing expression of kidney injury molecule-1 (Kim-1) in the kidney sections. **(B)** Quantification data presented as mean fluorescence staining showing increased expression of Kim-1 in the kidneys of DOCA-salt treated hypertensive mice compared with sham treated wild-type (WT) mice. This increase in Kim-1 expression was blunted in B1R deficient mice (B1RKO) mice with DOCA-salt treatment (*n* = 5, Two-way ANOVA, *P < 0.01 vs. Sham, ^†^*P* < 0.05 vs. WT + DOCA). **(C)** At the end of 3 weeks of DOCA-salt treatment, urinary albumin to creatinine levels ratio was significantly increased in WT mice, which was prevented in B1RKO mice (*n* = 6, Two-way ANOVA, *P < 0.01 vs. Sham, ^†^*P* < 0.05 vs. WT + DOCA).

Kidney injury molecule-1 (Kim-1) is an early clinical biomarker of acute injury, but it has also recently been utilized as an indicator of chronic kidney tissue damage (33). In WT control mice, there was little expression of Kim-1; however, DOCA-salt treatment induced a marked upregulation of Kim-1 (Figure 4A), which was confirmed by quantification data (Figure 4B; *p* < 0.01). In B1RKO mice, the level of Kim-1 was not upregulated by DOCA-salt treatment, indicating a protective role of B1R knockdown in kidney injury. Additionally, albumin-to-creatinine ratio is often used to detect albuminuria, which is a marked elevation in urinary albumin levels and a known biomarker of kidney damage. As an additional indicator of kidney injury, albumin-to-creatinine ratio was significantly elevated in the urine because of DOCA treatment in WT mice; however, this effect was not observed in B1RKO mice (Figure 4C; *p* < 0.01).

### B1R Gene Deletion Prevents Inflammation in the Kidney During DOCA-Salt Hypertension

**Figure 5:**
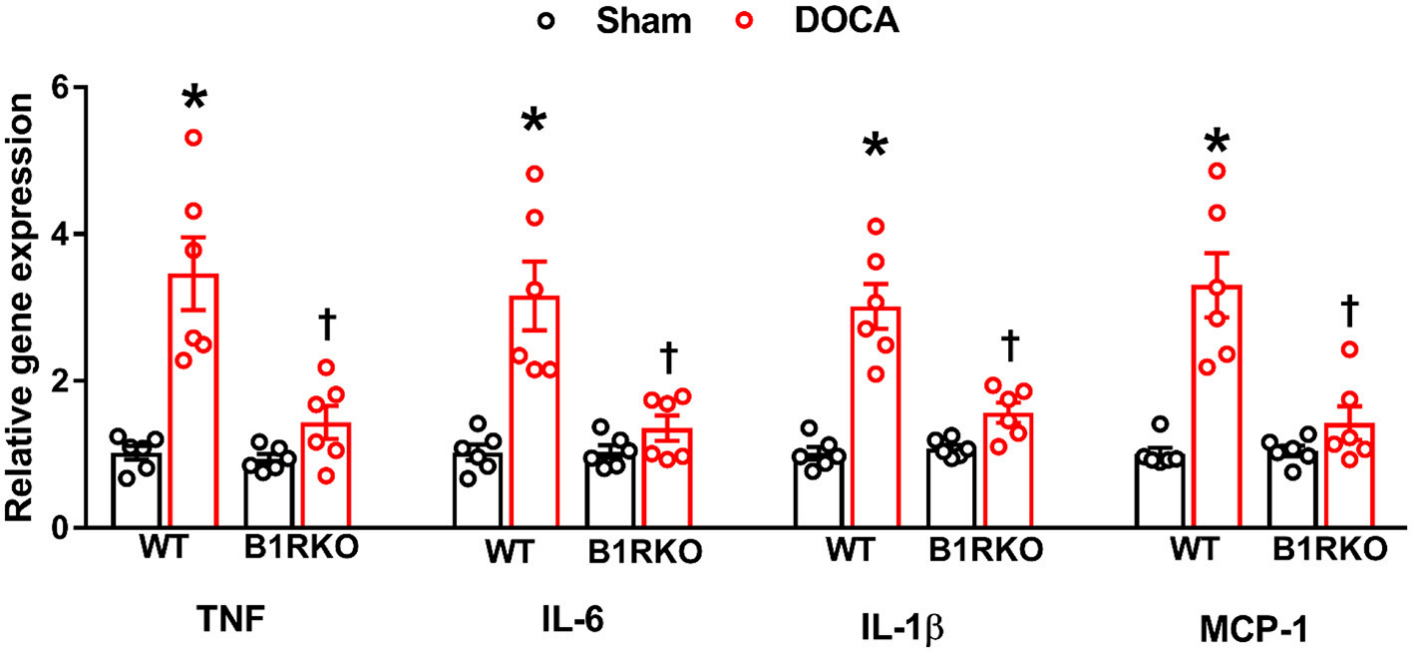
B1R gene deletion prevents inflammation in the kidney during DOCA-salt-induced hypertension. DOCA-salt treatment for 3 weeks significantly increased mRNA expression of pro-inflammatory cytokines TNF, IL-6, IL-1β and chemokine MCP-1 in the kidney cortex of wild-type (WT) mice compared with sham mice. B1R knockout (B1RKO) mice did not show this DOCA-salt-induced increase in inflammatory markers. Gene expression measured by real time RT-PCR. Data was normalized to β-actin and presented as mean ± SEM (*n* = 6, Two-way ANOVA, *P < 0.01 vs. Sham, ^†^*P* < 0.05 vs. WT + DOCA).

Although inflammation at acute time points is beneficial in tissue repair and healing, chronic systemic inflammation is also associated with increased mortality in patients (34). Previous studies have shown that impaired renal functioning prolongs the plasma t_1/2_ of several proinflammatory cytokines (35, 36). Here we measured the gene expression of pro-inflammatory cytokines and chemokines in the kidney and found a significant elevation in TNF, IL-6, IL-1β, and MCP-1 caused by DOCA-salt treatment in WT mice. However, this effect was blunted in B1RKO mice (Figure 5; *p* < 0.01).

### B1R Is Expressed in Human Kidney Proximal Tubular Epithelial Cells

**Figure 6:**
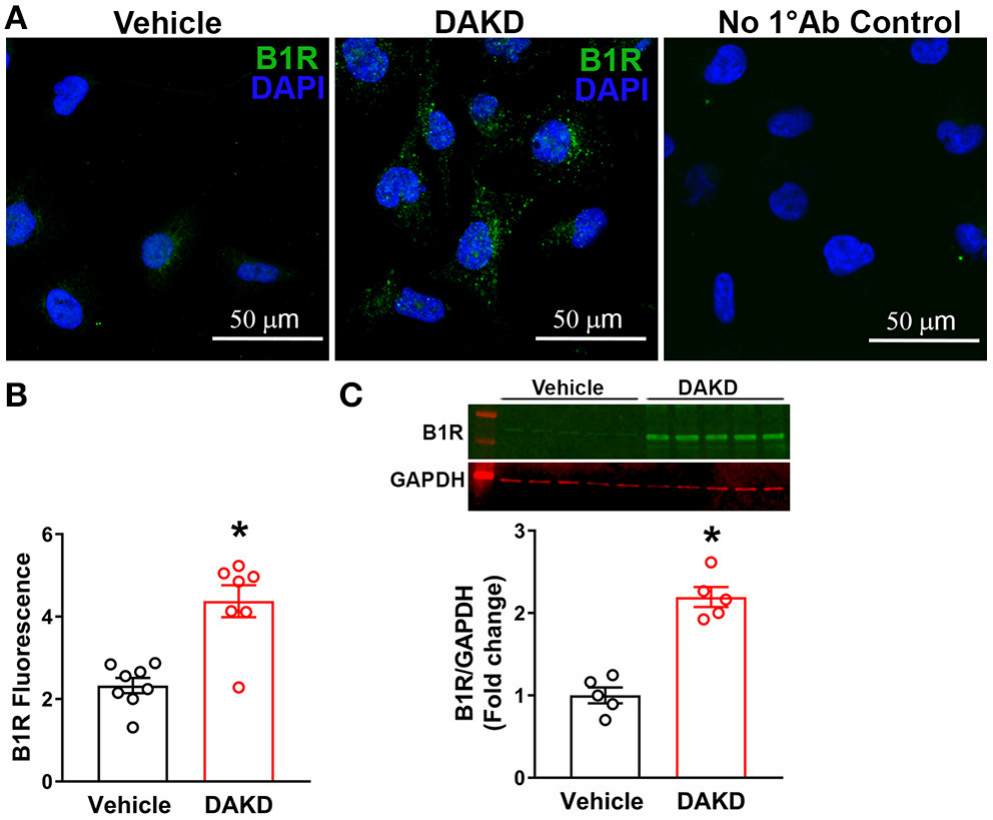
B1R expression in human kidney proximal tubular epithelial (HK-2) cells. **(A)** Representative photomicrographs showing immunofluorescence of B1R in HK-2 cells treated vehicle or B1R specific agonist DAKD for 24 h. Control HK-2 cells without primary antibody (No 1°Ab control) incubation and with only secondary antibody incubation shows no staining. **(B)** The quantification of B1R immunoreactivity shows that the DAKD treatment of HK-2 cells for 24 h significantly upregulated the B1R expression (*n* =7–8, Unpaired, 2-tailed *t*-test, *P < 0.01 vs. Vehicle). **(C)** Representative western blot image and quantification data showing significantly increased B1R protein expression in HK-2 cells following stimulation with DAKD for 24 h (*n* = 5, Unpaired, 2-tailed *t*-test, *P < 0.01 vs. Vehicle).

To further understand the direct role of B1R activation in the kidney, we used cultured human proximal tubular epithelial cells (HK-2) and treated them with B1R specific agonist DAKD. Immunofluorescence staining indicated that at baseline levels, B1R was not substantially expressed in HK-2 cells (Figure 6A), but stimulation with DAKD (300 nM) for 24 h significantly increased B1R expression as evidenced by the fluorescence quantification data (Figure 6B; *p* < 0.01). This B1R immunostaining was confirmed to be specific to B1R upon validation with a secondary antibody only staining as a negative control. We further confirmed this B1R protein induction in HK-2 cells by western blot analysis showing significantly increased B1R protein expression following stimulation with DAKD for 24 h (Figure 6C; *p* < 0.01). In addition, stimulation of HK-2 cells with DAKD for 24 h was shown to increase the immunoreactivity of α-SMA, fibronectin and Kim-1 (Supplementary Figure 2) suggesting a direct evidence that the activation of B1R can indeed modulate the fibrogenic phenotype and can lead to tissue injury.

### Blocking B1R, Not B2R Reduces Oxidative Stress in the Mice Kidney and HK-2 Cells

**Figure 7:**
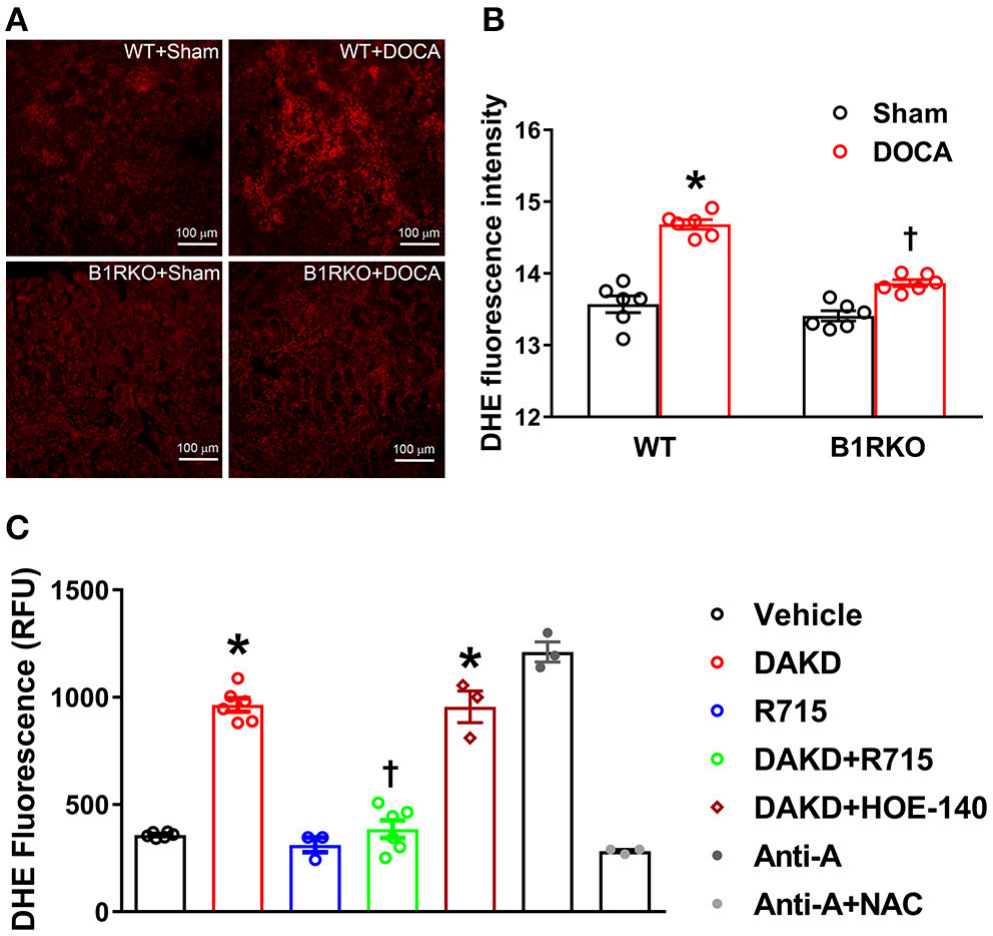
B1R blockade reduces reactive oxygen species (ROS) production in the kidney and HK-2 cells. **(A)** Representative photomicrographs showing dihydroethidium (DHE) staining in the kidney sections. **(B)** DHE staining quantified as relative fluorescence intensity showing increased superoxide generation in the kidneys of DOCA-salt treated hypertensive mice compared with sham treated wild-type (WT) mice. This increase in DHE staining was blunted in B1R deficient mice (B1RKO) mice with DOCA-salt treatment (*n* = 6, Two-way ANOVA, *P < 0.01 vs. Sham, ^†^*P* < 0.05 vs. WT + DOCA). **(C)** ROS production measured by microplate DHE assay showing B1R specific agonist DAKD stimulation of HK-2 cells induced a significant increase in total ROS production indicating increased oxidative stress, and pre-treatment with a specific B1R antagonist (R715, 10 μM) prevented this DAKD-induced ROS production. Pretreatment with specific B2R antagonist (HOE-140, 10 μM) for 1 h was not able to prevent the ROS production suggesting the B1R specific ROS production (*n* = 6 independent culture wells/group). Treatment with antimycin A (Anti-A), an inhibitor of complex III of the mitochondrial electron transport chain, is used as a positive control for ROS generation and treatment with N-Acetyl-L-Cysteine (NAC) is used as an antioxidant control. Statistical significance: One-way ANOVA followed by Tukey's multiple comparisons test. *p < 0.05 compared to vehicle, ^†^*p* < 0.05 compared to Ang II (*n* = 3–6, One-way ANOVA, *P < 0.01 vs. Vehicle, ^†^*P* < 0.05 vs. DAKD).

ROS such as super oxide production can lead to oxidative stress and are implicated in hypertension-induced renal damage (37). Recent studies have indicated that the progression of CKD is further propagated by an elevation in superoxide production and oxidative stress mediators (38). Therefore, we determined whether B1R blockade can prevent hypertension induced oxidative stress in the kidney. We utilized DHE staining as an indicator of superoxide production contributing to oxidative stress. In sham treated WT mice and B1RKO mice, there was no difference in the fluorescence intensity of DHE staining in kidney sections. In contrast, DOCA-salt treatment significantly increased DHE fluorescence intensity in WT mice kidneys, which was attenuated in B1RKO mice kidneys (Figures 7A,B). To specifically investigate the role of B1R activation, we used the HK-2 cells to directly activate B1R and determine its effects on superoxide production using a plate-based DHE fluorescence assay. We first confirmed our assay specificity by treating the cells with antimycin A as a positive control, which significantly increased DHE fluorescence indicating increased superoxide generation. Additionally, pre-treatment with a ROS inhibitor NAC was able to prevent the antimycin A-induced DHE fluorescence suggesting the assay was specific to detect the ROS production. HK-2 cells treated with B1R agonist DAKD showed a significant increase in DHE fluorescence intensity indicating increased superoxide production (Figure 7C). However, B1R blockade by its antagonist R715 attenuated this superoxide production, suggesting B1R plays an important role in kidney injury through oxidative damage. Additionally, using a B2R specific antagonist HOE-140, we were able to further establish that the attenuation of superoxide production by R715 was directly due to B1R, not mediated by B2R.

### B1R Expression Is Upregulated and Correlated With Collagen Expression in Human Kidneys of Hypertensive Chronic Kidney Disease

**Figure 8:**
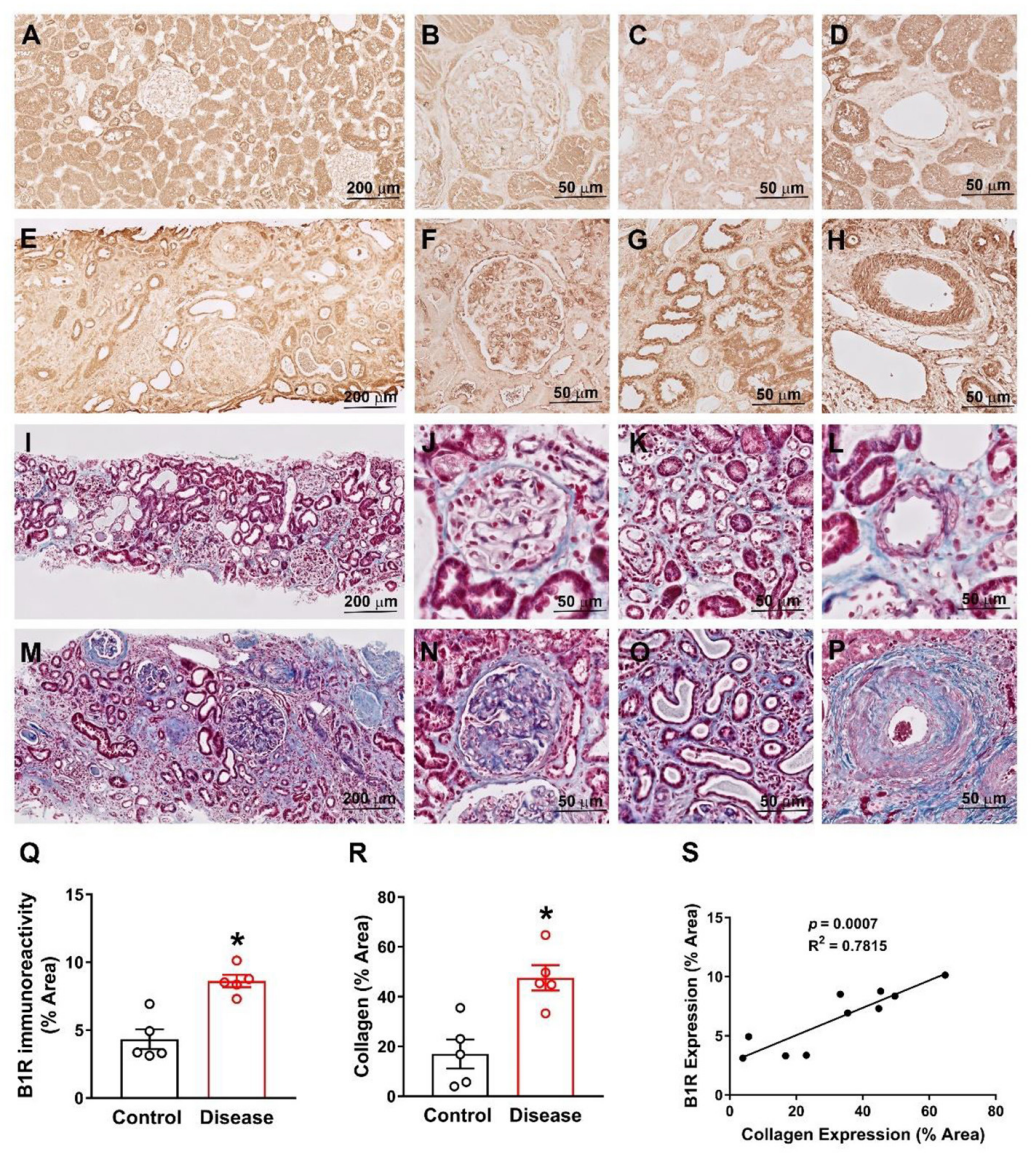
B1R expression is increased and correlates with collagen expression in human kidney sections from hypertensive chronic kidney disease. Representative immunohistochemistry photomicrographs showing B1R specific immunoreactivity in kidney sections of healthy controls **(A)**, specifically in glomerulus **(B)**, tubules **(C)** and arteries **(D)**. The B1R specific immunoreactivity is increased in kidney sections from renal disease **(E)**, specifically in glomerulus **(F)**, tubules **(G)** and arteries **(H)**. Representative photomicrographs showing Masson Trichrome staining for collagen deposition in health control kidney section **(I)** with in glomerulus **(J)**, tubules **(K)** and arteries **(L)**. The collagen deposition was increased in kidney sections from the kidney disease subjects **(M)**, specifically in glomerulus **(N)**, tubules **(O)** and arteries **(P)**. Quantification data showing increased B1R **(Q)** and collagen **(R)** expression in kidney sections from renal disease subjects (*n* = 5, Unpaired, 2-tailed *t*-test, *P < 0.01 vs. Control). B1R immunoreactivity is positively correlated with collagen deposition **(S)**. Pearson correlation and least-squares linear regression were used to determine correlation coefficients between variables.

We first confirmed that B1R is expressed in human kidneys as evident from the immunohistochemical staining of B1R in human kidney sections (Figures 8A–H). Then, we compared B1R expression in kidneys from the control group with the patient group (see methods). The expression of B1R was higher in the glomeruli, tubules, and arterioles in kidneys of patients compared to that of controls (Figures 8A–H, quantitative data in Figure 8Q). Similarly, Masson's Trichrome staining showed increased fibrosis as indicated by increased collagen deposition in the kidney sections of hypertensive chronic kidney disease patients when compared to healthy controls (Figures 8I–P, quantitative data in Figure 8R). To determine whether B1R levels in the kidney are significantly correlated with fibrosis, we performed linear regression analysis. Our results showed that there is a statistically significant correlation of B1R expression with fibrosis in human kidney sections (Figure 8S).

## Discussion

A large body of experimental and physiological evidence indicates that fluid control and renal perfusion pressure are mainly involved in maintaining the arterial circulation and blood pressure (7, 24). Alarmingly, more than 20% of hypertension patients will further develop CKD. Given this statistic, it is necessary to further understand the pathogenesis of hypertensive kidney disease to halt its progression. B1R has been shown to be directly involved in inflammation and oxidative stress. Interestingly, the gene and protein expression of B1R in the kidneys has been shown to be elevated in hypertensive models; however, there have been no studies to indicate its role in pathogenesis hypertensive end organ damage (16–18). Our study is the first, to our knowledge, to investigate whether B1R knockdown prevents hypertension-induced kidney injury.

Previously, our laboratory showed that B1R contributes to neurogenic hypertension through centrally activated mechanisms (13). In this study, we build upon these findings by revealing an elevation in renal B1R gene and protein expression within the kidneys in DOCA-salt hypertension. Since the kidney is a complex organ system, it can be further subdivided into the components of the nephron which include the glomeruli, tubules, and the collecting ducts. To visualize B1R expression, we used immunofluorescence staining to determine DOCA-salt induced an increase in B1R expression within the glomeruli and tubules of the kidney (Figure 1C). Glomerular injury and tubulointerstitial damage are some of the key hallmarks of progressive kidney disease, and it is established that chronic inflammation is one of the primary drivers of these enduring injuries (39). Given the implications of B1R in inducing chronic inflammation, the increased B1R expression in the glomerulus and tubules in the kidneys of DOCA-salt treated mice further support the evidence that B1R plays a role in hypertensive kidney injury and remodeling.

For many chronic pathological conditions, fibrosis is considered the ultimate outcome. Although collagen deposition is an essential, yet typically, reversible part of tissue recovery, normal tissue repair can progressively evolve into a maladaptive and irreversible fibrotic response if the tissue is repetitively injured. Fibrosis is characterized by the excessive deposition of collagens and the accumulation of fibrous connective tissue. Fibroblasts are a common cell type found in the body and are known to secrete collagen proteins that are used to maintain a structural framework for many tissues. When injured, fibroblasts are stimulated and undergo differentiation into myofibroblasts, and this process is one of the major contributing factors in extracellular matrix deposition and fibrosis (40, 41). TGF-β activation is well-known for its ability to induce fibrotic changes and is consistently observed in experimental models of fibrosis (42). Additionally, myofibroblasts are commonly identified by the expression of α-SMA (43). To determine whether B1R plays a role in fibrosis, we investigated levels of fibrosis markers in WT and B1RKO mice subjected to the DOCA-salt paradigm. We observed the gene expression of profibrotic markers such as collagen I/IV, MMP-9, TIMP-1, TGF-β, fibronectin, α-SMA, and CTGF were all increased in WT + DOCA mice, but this effect was attenuated in B1RKO mice. Fibrosis is defined by the deposition of ECM proteins; thus, we quantified fibronectin a commonly measured ECM glycoprotein. Using immunofluorescence, we showed that α-SMA, TGF-β, and fibronectin protein expression was significantly increased in hypertensive animals (WT + DOCA) indicating an increased presence of myofibroblasts and ECM proteins, which was attenuated in B1RKO mice. This evidence suggests that B1R knockdown prevented the transdifferentiation of renal fibroblast to myofibroblast in hypertension.

Next, we wanted to investigate clinical biomarkers in our model that are often used to define kidney injury. Kim-1 is a type 1 transmembrane protein, with an immunoglobulin and mucin domain, whose expression is markedly upregulated in response to proximal tubular injury (44). Using this clinical biomarker, we saw a significant elevation in Kim-1 expression in WT + DOCA treated mice, and this effect was attenuated in B1RKO + DOCA mice. Furthermore, we showed that WT + DOCA mice have a greater albuminuria, and B1RKO mice have an attenuation of this increase suggesting B1R possibly has a role in glomerular injury as well. In addition, direct stimulation of B1R in HK-2 cells induced the expression of α-SMA, fibronectin and Kim-1 suggesting the direct involvement of B1R in injury to the kidney tubules. The results indicate that B1R blockade is a potential strategy for preventing kidney injury.

In recent years, inflammation and oxidative stress have been investigated as primary drivers of many chronic pathologies. Typically, patients with CKD suffer from a severely impaired antioxidative system, which extenuates the severity of their renal impairments (45). Previously, our laboratory showed that the B1R blockade can attenuate elevated oxidative stress and proinflammatory markers (13, 28). In line with these findings, we found that B1R knockdown mitigated renal inflammation by preventing an upregulation in the gene expression of TNF, IL-6, IL-1β, and MCP-1 in the kidney. Given previous studies indicating an increased plasma t_1/2_ of proinflammatory cytokines in renal impairment, we provide corresponding evidence that B1R knockdown not only prevents inflammation and oxidative stress but also reduces markers associated with renal injury.

Increasing evidence has begun to suggest that oxidative stress has become a major contributing factor in kidney injury/damage and chronic kidney disease (46). We have shown previously that treatment with B1R specific antagonist R715 reduced superoxide production and NADPH (NOX) oxidase 2 and 4 gene expression in primary neurons (28). In this study we showed that DHE staining highlighted a significant increase in superoxide production in WT + DOCA mice, which was attenuated in B1RKO + DOCA mice. Together, these results demonstrate that blocking B1R avert fibrosis, oxidative stress, and inflammation in the kidneys of hypertensive mice. The results of our study highlighted the advantageous effects of B1R gene deletion in completely preventing kidney injury, inflammation, and oxidative stress in a hypertensive renal injury model. A previous study demonstrated that B1R antagonism is equally efficient as angiotensin receptor 1 antagonism in reducing renal fibrosis in experimental obstructive nephropathy (47). However, in the present study we have not investigated the involvement of other key mediators of hypertension such as renin-angiotensin-aldosterone system and the endothelin receptors. Therefore, further studies are needed to delineate the interaction of B1R with the other key systems involved in hypertensive chronic kidney disease.

To further establish if these findings are applied to human kidneys, we utilized human epithelial proximal tubular cells (HK-2) and activated them with the endogenous ligand of B1R, DAKD. We showed that DAKD treatment visibly increased B1R expression and also increased fibronectin and Kim-1 expression in treated HK-2 cells. Using a plate based DHE assay, we showed that B1R agonism resulted in an increase in superoxide production. Interestingly, using HOE-140 (B2R specific antagonist), we were unable to see an attenuation in the production of ROS. However, while pretreating HK-2 cells with R715 (B1R specific antagonist), we were able to block the production of ROS. Thus, our evidence further provides us with confidence that the effects are solely due to the B1R and not a compensatory mechanism of the B2R.

An important finding from our study is that B1R immunoreactivity is significantly higher in the hypertensive chronic kidney disease patients compared to normal healthy controls, indicating the potential clinical significance of B1R upregulation in the kidney. In addition, this increase in B1R expression was observed within the glomeruli, tubules, and arteries. To determine a further causational effect of elevated B1R expression with pathology, we stained the same human kidney samples with masson's trichrome to determine fibrosis levels as evidenced by collagen deposition. Comparable to the B1R staining, the diseased population exhibited an elevated collagen deposition. We also found a positive correlation between B1R expression and collagen deposition. Thus, our data suggest that the increase in B1R expression is associated with elevated fibrosis in hypertensive chronic kidney disease patients.

Overall, the major findings of our present study are that (1) DOCA-salt treatment increases B1R gene and protein expression in the kidney; (2) B1R knockdown reduces the gene and protein expression of DOCA-salt-induced profibrotic and proinflammatory markers; (3) B1R knockdown reduces clinical markers of kidney damage which are elevated by DOCA treatment; (4) Human proximal tubular cells (HK-2) show an elevation of B1R when treated with B1R agonist (DAKD), and this effect is rescued by B1R antagonist (R715); (5) Both *in vivo* and *in vitro* studies show that B1R protects against superoxide production and oxidative stress in our model; (6) Hypertensive human patients show a significantly elevated B1R expression which is correlated with increased fibrosis.

One limitation of our study is that we only used male mice in our animal experiments. Using both males and females will be necessary to further confirm any sex differences in DOCA-salt treatment and the Kinin B1R expression in the kidney. Another limitation of our study is the small human sample size. The samples were scored for glomerular injury and tubulointerstitial damage by an independent pathologist to correctly identify them within their respective groups. Despite the small sample size, we were able to establish significant differences in B1R immunoreactivity and collagen deposition, and their correlation within the control and diseased groups. Increasing sample size and further differentiating sex differences will be a future goal in both human and animal studies.

The mainstay of the current reno-protective measures target the angiotensin pathway through ACEi/ARB to inhibit the angiotensin II mediated kidney fibrosis. Despite widespread use of ACEi/ARB, hypertensive kidney disease remains one of the leading causes of end-stage renal disease (ESRD) and acts as a massive clinical burden. It is important to elucidate the pathogenesis of hypertensive kidney disease, particularly if there is any non-angiotensin II mediated pathways, to treat and improve prognosis of the disease. Our results show that B1R may potentially be a novel therapeutic target to prevent the progression of the disease. B1R inhibitors can be a promising alternative reno-protective agent, overcoming the shortcomings of the ACEi/ARB. Our study did not clearly elucidate the exact mechanisms of kidney injury induced by B1R. Further studies are needed to understand detailed mechanisms and the localization of B1R induced effects.

## Data Availability Statement

The original contributions presented in the study are included in the article/Supplementary Material, further inquiries can be directed to the corresponding author/s.

## Ethics Statement

Ethical review and approval was not required for the study on human participants in accordance with the local legislation and institutional requirements. Written informed consent for participation was not required for this study in accordance with the national legislation and the institutional requirements. The animal study was reviewed and approved by East Carolina University Animal Care and Use Committee.

## Author Contributions

SS conceptualized and designed research. DB, RP, AW, and SS performed the experiments, collected data, analyzed data, and prepared figures. AT contributed to human kidney sections staining and interpretation of the results. DB, RP, and SS wrote the manuscript. All authors approved the final version of the manuscript.

## Funding

This study was supported by the National Heart, Lung, and Blood Institute of the National Institutes of Health under award number 5R01HL153115 (SS).

## Conflict of Interest

The authors declare that the research was conducted in the absence of any commercial or financial relationships that could be construed as a potential conflict of interest.

## Publisher's Note

All claims expressed in this article are solely those of the authors and do not necessarily represent those of their affiliated organizations, or those of the publisher, the editors and the reviewers. Any product that may be evaluated in this article, or claim that may be made by its manufacturer, is not guaranteed or endorsed by the publisher.
