# Supplementary material for: Kinin B1 Receptor Mediates Renal Injury and Remodeling in Hypertension
Source: Front Med (Lausanne). 2022 Jan 18;8:780834. doi: 10.3389/fmed.2021.780834 (PMC8804098; doi:10.3389/fmed.2021.780834)
Supplement: Supplementary file 1 [file Presentation_1.PPTX]

## Slide 1
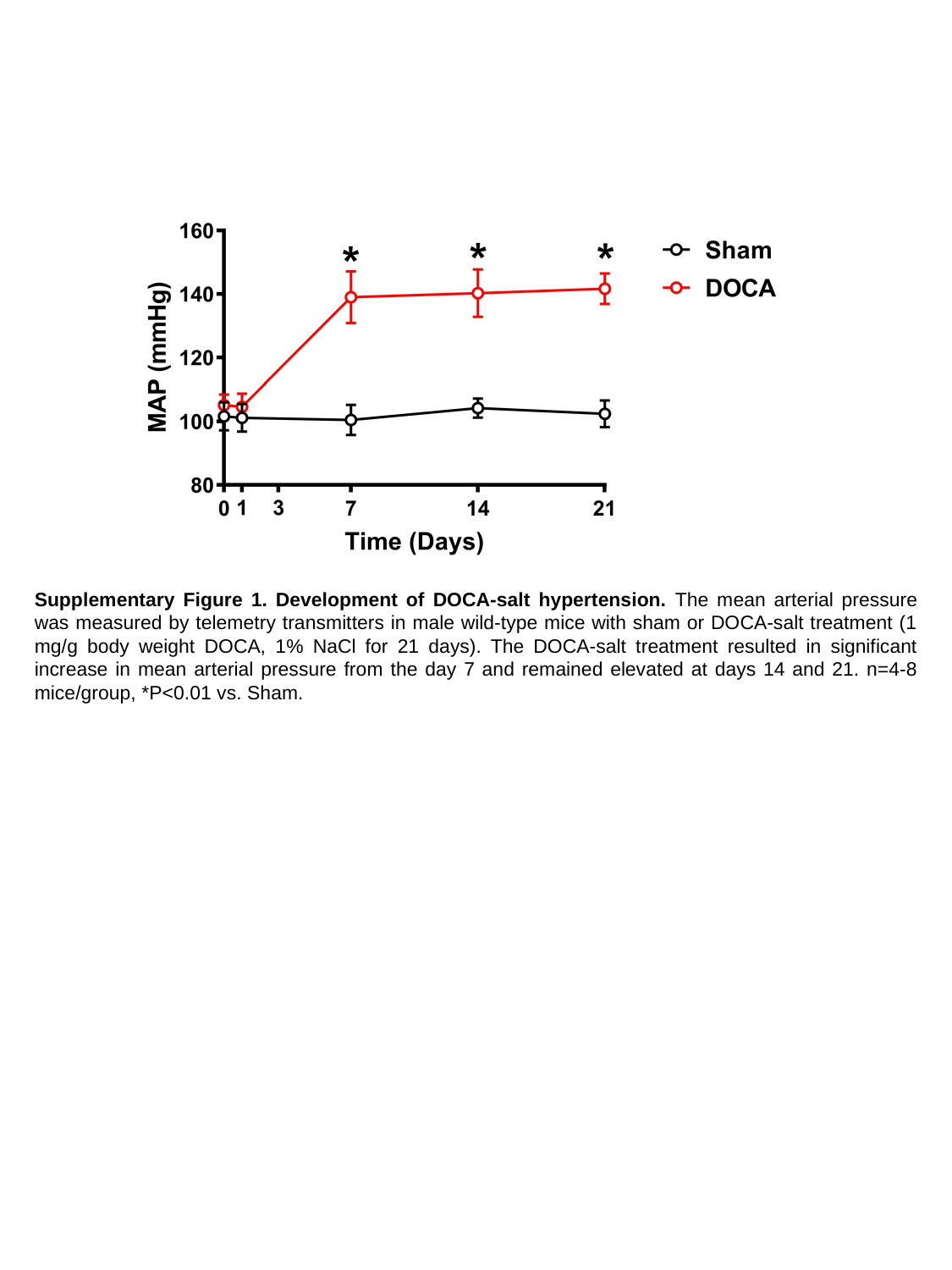

Supplementary Figure 1. Development of DOCA-salt hypertension. The mean arterial pressure was measured by telemetry transmitters in male wild-type mice with sham or DOCA-salt treatment (1 mg/g body weight DOCA, 1% NaCl for 21 days). The DOCA-salt treatment resulted in significant increase in mean arterial pressure from the day 7 and remained elevated at days 14 and 21. n=4-8 mice/group, *P<0.01 vs. Sham.

## Slide 2
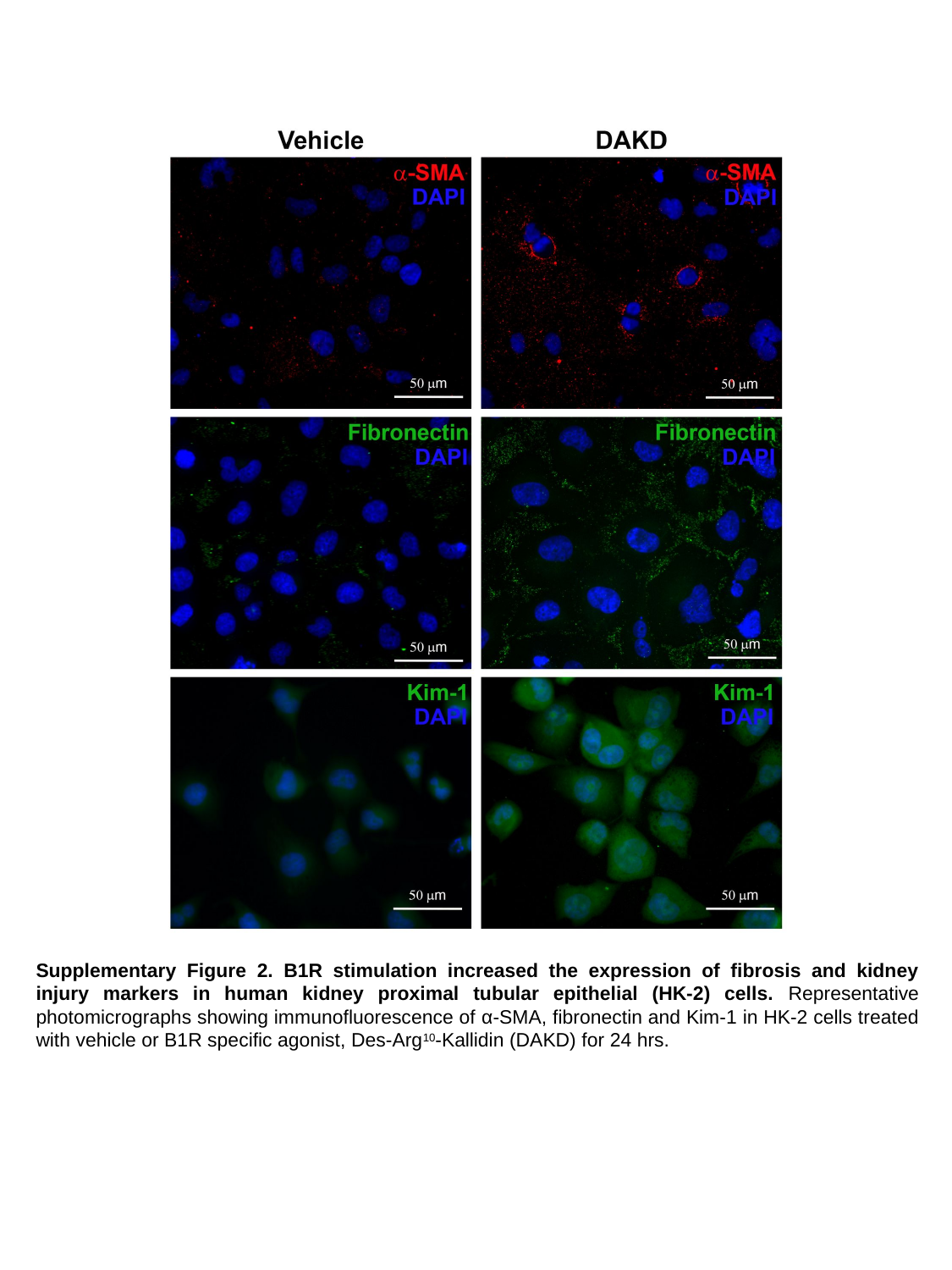

Supplementary Figure 2. B1R stimulation increased the expression of fibrosis and kidney injury markers in human kidney proximal tubular epithelial (HK-2) cells. Representative photomicrographs showing immunofluorescence of α-SMA, fibronectin and Kim-1 in HK-2 cells treated with vehicle or B1R specific agonist, Des-Arg10-Kallidin (DAKD) for 24 hrs.
